# Social sensing of urban land use based on analysis of Twitter users’ mobility patterns

**DOI:** 10.1371/journal.pone.0181657

**Published:** 2017-07-19

**Authors:** Aiman Soliman, Kiumars Soltani, Junjun Yin, Anand Padmanabhan, Shaowen Wang

**Affiliations:** 1 CyberGIS Center for Advanced Digital and Spatial Studies, National Center for Supercomputing Applications, University of Illinois at Urbana-Champaign, Champaign, Illinois, United States of America; 2 Illinois Informatics Institute, University of Illinois at Urbana-Champaign, Champaign, Illinois, United States of America; 3 Department of Geography and Geographic Information Science, University of Illinois at Urbana-Champaign, Champaign, Illinois, United States of America; Stony Brook University, Graduate Program in Public Health, UNITED STATES

## Abstract

A number of recent studies showed that digital footprints around built environments, such as geo-located tweets, are promising data sources for characterizing urban land use. However, challenges for achieving this purpose exist due to the volume and unstructured nature of geo-located social media. Previous studies focused on analyzing Twitter data collectively resulting in coarse resolution maps of urban land use. We argue that the complex spatial structure of a large collection of tweets, when viewed through the lens of individual-level human mobility patterns, can be simplified to a series of key locations for each user, which could be used to characterize urban land use at a higher spatial resolution. Contingent issues that could affect our approach, such as Twitter users’ biases and tendencies at locations where they tweet the most, were systematically investigated using 39 million geo-located Tweets and two independent datasets of the City of Chicago: 1) travel survey and 2) parcel-level land use map. Our results support that the majority of Twitter users show a preferential return, where their digital traces are clustered around a few key locations. However, we did not find a general relation among users between the ranks of locations for an individual—based on the density of tweets—and their land use types. On the contrary, temporal patterns of tweeting at key locations were found to be coherent among the majority of users and significantly associated with land use types of these locations. Furthermore, we used these temporal patterns to classify key locations into generic land use types with an overall classification accuracy of 0.78. The contribution of our research is twofold: a novel approach to resolving land use types at a higher resolution, and in-depth understanding of Twitter users’ location-related and temporal biases, promising to benefit human mobility and urban studies in general.

## Introduction

Characterizing urban land use is becoming increasingly important because, by 2050, it is estimated that 66% of the world’s population will live in urban areas [1]. Traditional sources of urban land use information, such as on-site survey and questionnaires, are costly, time-consuming, and limited to a small number of human subjects. Previous research showed that high-resolution remote sensing data can be used to aid the process of mapping urban environments. However, urban mapping using remotely-sensed data is limited to monitoring land cover since land utilization is difficult to infer from physical infrastructure–specifically in mixed urban environments. On the other hand, the traditional perception of urban land use is rapidly changing due to the introduction of the Internet. For example, a residential place could function as a location for employment or education thanks to the power of networked communications. Given the increase in interest to manage cities in smarter ways, there is a critical need for a low-latency land use observations that complement conventional survey and remote sensing data.

During the past decade, digital footprints within urban environments have grown exponentially. Every day, massive amounts of geo-tagged information are generated via photo and video sharing platforms (Flickr, YouTube, Facebook, etc.), and micro-blogging services (Twitter and Foursquare) [2–4]. Although these big data streams were not initially intended to be sources of geospatial information, they provide a valuable lens on how people interact with their urban space, which complements authoritative geospatial data sources [5, 6]. However, the exponential increase in such big data combined with a lack of spatial structure makes data synthesis a challenge [7–10]. As a result, spatial data synthesis methods for heterogeneous data streams is an active area of research [11]. In this research, we investigate geo-located Twitter data for the purpose of characterizing urban land use types. We selected the City of Chicago as a case study because of the availability of updated authoritative land use and travel survey datasets.

## Related work

Previous research on characterizing urban land use using Twitter data focused on analyzing changes in the number of tweets sent from a geographic location over time. For example, Vanessa et al. [12] extracted hourly changes in the number of tweets during weekdays and weekends and used them to group urban regions in New York, London, and Madrid, based on temporal tweeting patterns. While Wakamiya et al. [13] mapped neighbors in Japanese cities based on relative changes in the number of tweets, number of unique users, and users’ movements during six-hour intervals. In addition to temporal activity, the context of tweets was used to infer land use types. For instance, Abbasi et al. [14] used a Latent Dirichlet Allocation algorithm to associate keywords related to six urban activities to geographic locations. Furthermore, information contained in users’ points of interest (POI) was found useful in mapping urban land use. For example, Zhan et al. [15] used Foursquare check-in data to extract seven temporal patterns of check-in activities, which were found to be associated with different land use types. Also, POIs from Foursquare were combined with OpenStreetMap data to delineate land use types at the parcel level for China [16]. Similar results were found by synthesizing POIs with Landsat images to produce a parcel-level land use map for the city of Beijing [17].

Although the aforementioned studies provided promising results, a remaining challenge is to define an objective space partition scheme prior to extracting Twitter temporal signatures. One could consider this challenge as a manifestation of the Modifiable Area Unit Problem (MAUP) [18, 19], where a change of geographic partitioning scheme would alter the statistical attributes of partitions. For example, Vanessa et al. [20] used a self-organizing map algorithm to segment urban areas. Similarly, Wakamiya et al. [21] found that both grid and administrative boundaries do not reflect the distribution of Twitter data. Instead, the authors used an Expectation Maximization algorithm to divide the urban space without defining the number of divisions in advance. Although statistical techniques reduced the uncertainty associated with applying subjective space partitioning schemes, the statistically-driven divisions often result in coarse spatial resolutions maps, which limits their potential to be integrated with other high-resolution data (e.g. land use maps based on remote sensing data). An alternative approach to characterize land use using Twitter data is analyzing user activities at the individual level. This alternative approach is desirable for the analysis of spatial patterns of geo-located tweets because human mobility research has shown that movements of an individual are predictable [22], universal among people from different socio-economic strata [23], and can be explained by geographically distinguishable locations (e.g. home, work, etc.) [24]. Moreover, research findings suggest that these mobility patterns are common among the Twitter user community [25].

In general, this research argues that analysis of Twitter users’ mobility patterns at the individual level would yield reliable information about urban land utilization. Our specific aims are a) characterize urban land use at an unprecedented spatial resolution by analyzing Twitter users’ activities at their key locations; b) avoid the limitations of dependence on a space-partitioning technique. We recognized that contingent issues, such as Twitter users’ biases to tweet from certain land use types, need to be examined before mining Twitter data. We tested detailed hypotheses about a) the spatial relations between Twitter users’ key-locations and land use parcels; b) Twitter users’ tendency to tweet from certain land use types and its impact on the representativeness of Twitter data of urban land use composition, and c) reliability of extracting land use types using temporal signatures of individual Twitter users. We tested our hypotheses using Twitter data and two independent datasets: the land use inventory for Northeastern Illinois [26] and the Chicago Travel Tracker Household Inventory [27].

## Conceptual framework

We introduce four basic scenarios to illustrate our hypotheses and assumptions about the mobility patterns of social media users and in particular those of Twitter users. Following, we tested each of these scenarios using a collection of Twitter data and ancillary variables to identify consistent patterns of social media users that could be used for characterizing urban land use.

**Random walker scenario**: In this scenario, the Twitter user moves randomly around the city and tweets only from new places (i.e. a random sample without replacement). For example, a tourist who tweets about new experiences. The geo-located tweets of a group of random walkers, when aggregated over a period of time, appear as a set of randomly distributed locations across the city.

**Preferential return scenario**: the phenomenon of preferential return postulates that people spend more than 90% of their time around a few key locations (home, work, etc.) [22] and that this behaviour is common among people from different demographics [23]. If a Twitter user exhibits this behaviour, her/his tweets would appear in clusters around the key locations of this user, particularly when accumulated over a long period. If we would arrange these key locations, for each user independently, in a descending order based on the number of tweets, we would expect that the rank of each cluster will be proportional to the time spent at that location.

**Semantic coherence scenario**: Although that the phenomenon of preferential return explains that the majority of peoples spend most of their time around a few key locations, it does not explain what to expect at these frequently visited locations. An implicit assumption that is often used, is that the top two locations are home and work locations for most of the people. If this assumption is applicable for Twitter users, the semantics (land use type) of top tweeted-from clusters could be inferred directly from their rank. A simple example is assigning the top tweeted-from location for any user as their home location [24, 28, 29].

**Temporal coherence scenario**: In this scenario, we associate each key location with a certain period of the day (e.g. morning, evening, etc.) based on the hours when the majority of tweets were posted. Although observing a Twitter user over a short period of time does not reveal the time windows of her/his key locations, the accumulation of tweets over a longer period of time is likely to indicate the time of the day that is associated with each location [25]. We also assume that the timing of tweets is dependent on the land use type at the cluster location and it is similar (coherent) for most of the users. If this assumption is true, classification algorithms could be applied to infer land use types of key locations based on the timing of tweets at each location.

## Results

### Preferential return of Twitter users

Although a density-based clustering algorithm, such as DBSCAN, can extract clusters of tweets (key locations) for each user independently, the extracted clusters might be artifacts and not necessarily associated with particular landmarks (more details about the selection of clustering parameters are given in the Methods section). We developed a spatial uncertainty index to quantify the degree of overlap between each cluster of tweets and the nearest land use parcel, which is the minimum mapping unit of the available land use map of Chicago [26]. The spatial uncertainty index was estimated by assigning each tweet in the cluster to the nearest parcel and calculating the relative weight of the most common land use parcel in the cluster. Therefore, a high index value indicates that all the tweets in the cluster are associated with the most common parcel.

**Fig 1:**
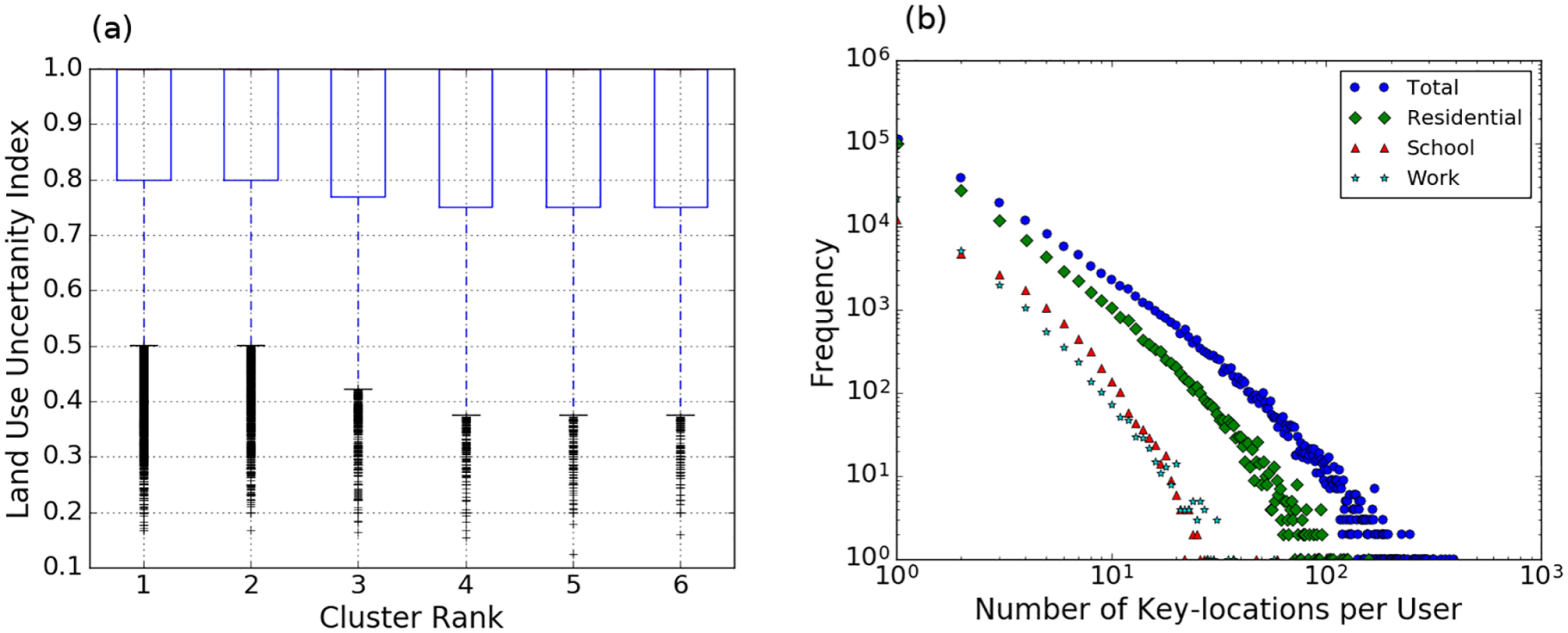
Spatial uncertainty. A: Box plots of the distribution of spatial uncertainty index grouped by rank; an index value of one indicates that all the tweets in a cluster are in the close proximity of a single land use parcel. Notice the strong left-skewed distribution, which indicates that the majority of the parcels are uniquely associated with a particular parcel. B: Log-log distribution of number of parcels per unique users grouped by activity types.

The distribution of spatial uncertainty for all clusters provides an evaluation metric of the overall overlap between users preferential return key locations and map parcels under the assumption that if clusters of tweets are artifacts then it is unlikely that each of them will be uniquely associated with a single parcel. The distribution of spatial uncertainty index for all clusters, grouped by cluster rank, is presented in (Fig 1a), where rank *one* designates the cluster with the largest number of tweets for each unique user. The box plot distribution shows a left-skewed distribution around one, where a value of one signifies that all the tweets in that cluster are in a close proximity with a single parcel. The interquartile range was between 1 and 0.75 for top ranks indicating that most of the identified clusters are uniquely associated with a land use parcel. Our results support that a large number of users do not follow a random walker scenario, rather they prefer to tweet from the vicinity of a few parcels as predicted by the phenomenon of preferential return.

Furthermore, we examined the number of key locations per user, particularly for land use types such as educational (e.g. schools and universities) and workplace (e.g. office). We assume that the possibility of relocation during the study period (2013-2016) is limited. This assumption is supported by our analysis of Twitter users’ engagement patterns, which revealed that the majority of users were engaged for less than a single year and their tweeting activities were usually concentrated within a few months (Figure 4 in S1 File). In this respect, Twitter data provide a short moving window to observe users’ activities, where older users continuously drop and new users are added.

The number of clusters per unique user reveals a heavy tail distribution, where the majority of users are associated with a single key location (Fig 1b). Nevertheless, the gradual decrease in the number of locations per user indicates that a number of users are associated with multiple schools, offices, and universities. This result is attributed to the fact that some land use parcels might contain multiple buildings. For example, a university campus parcel contains lecture halls, cafeterias, and parking lots. The combination of the high spatial resolution of geo-located Tweets and the fixed clustering algorithm parameters could result in resolving multiple key locations or break down a large irregular cluster located on the same parcel. Although the spatial uncertainty index captures the unique relationship between a single cluster and a land parcel, it does not grantee a one-to-one spatial relationship between them.

### Semantic coherence of Twitter users

An implicit assumption of the preferential return phenomenon is that the order of land use types of key user locations (e.g. home, work, and leisure) is similar for the majority of users, we refer to this assumption here by semantic coherence as it is discussed in the conceptual framework section. Moreover, it is often cited that rank one and two are the home and work locations for most of the users even without sufficient empirical evidence [30].

**Fig 2:**
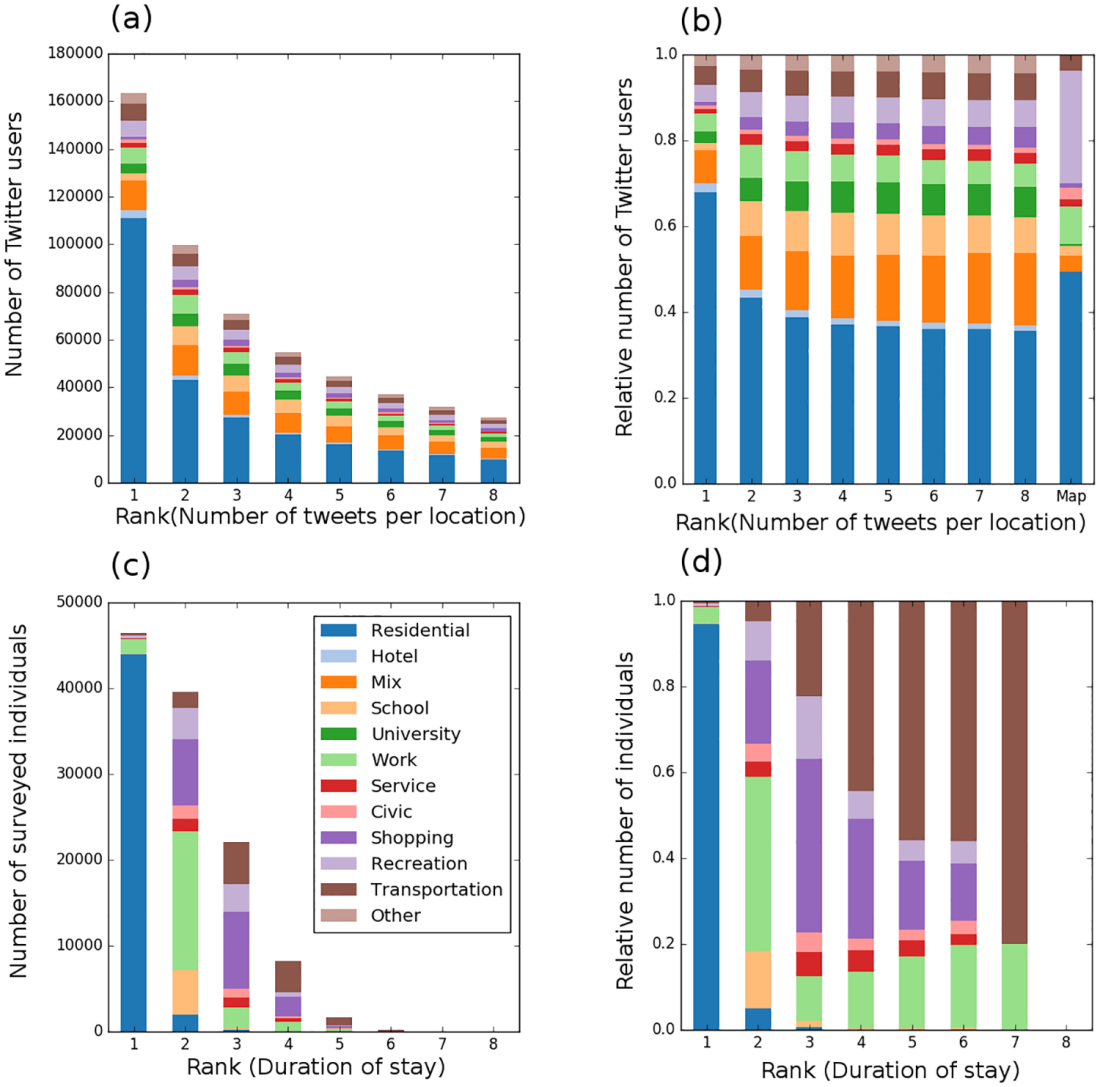
Semantics of top tweeted-from locations. A-B: Count of unique users grouped by land use types and ranks of their top ten key locations; absolute count (A) and normalized count (B). C-D: Count of surveyed individuals who reported their stay times at different locations during the day grouped by land use types and ranks (based on the duration of stay); absolute count (C) and normalized count (D). Data were extracted from the travel survey of Chicago and present an estimate of the preferential return of Chicago residents at the time of the survey.

We estimated the degree of semantic coherence among Chicago population using reported stay time at different land use types in the Travel Tracker survey of Chicago residents [27]. The reported land use types by each unique surveyed individual were ranked based on the duration of stay. (Fig 2c and 2d) shows the results from pooling the top staying locations for all surveyed individuals and group them by rank, where rank one is the location with the longest stay period. The results suggest a significant presence of semantic coherence among Chicago residents at least for the top location (rank one). For example, the longest time duration spent by more than ninety percent of Chicago residents is at home. However, the semantic coherence becomes more dependent on other factors, such as age and day of the week, starting from rank two, where probabilities of a person spending her/his time at work, school or shopping become equal.

Similarly, we examined the semantic coherence in Twitter users key locations by pooling common land use types for all users and group them by ranks based on the number of tweets. If users tweeting activities exhibit semantic coherence, we would expect that each rank is dominated by a specific land use type. On the contrary, our results reveal that rather than finding a common land use among the users for each rank, Twitter users’ preferences varied considerably as indicated by the combination of frequent land use types observed at each rank, refer to (Fig 2a and 2b). For example, residential land use accounted for 65% of top tweeted-from location. Remarkably, common land use types of Twitter users’ top locations were found to be correlated across ranks (Pearson correlation ranged between 0.92-0.99), which indicates a strong decoupling between common land use types and their ranks.

We tested the existence of a semantic coherence among Twitter users. We assumed that if a semantic coherence exists among Twitter users, their common land use types would resemble those common types which were reported by individuals in the travel survey. The comparison was conducted by measuring the dynamic time warping (DTW) similarity of common land use types of 200 random samples taken equally from Twitter and the survey records (100 samples each), where each sample is made of 10,000 individuals. In order to control for random effects, we introduced a control comparison of 100 samples of 10,000 random land use parcels each, which represents common land use types under absolute random selection and no semantic coherence.

**Fig 3:**
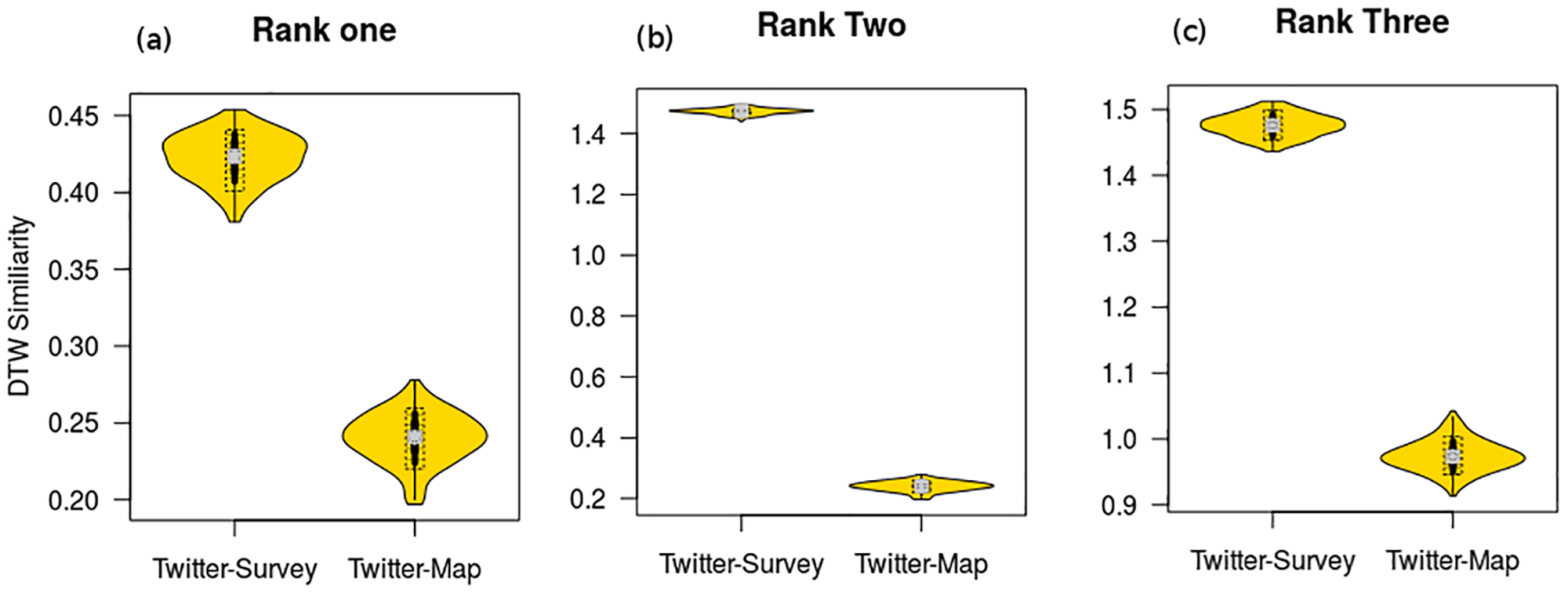
Similarity of common land use types among Twitter users and the Travel Tracker survey individuals. A-C: Violin plots of similarity of common land use types between Twitter users and the Travel survey individuals compared to the control group (a random sample from the land use map of Chicago for rank one (A), rank two (B) and rank three (C). Each sample is made of 10,000 individuals in case of Twitter and the travel survey and 10,000 random land use parcel in the case of the random map sample.

Our results (Fig 3) show that common land use types of Twitter users are more similar to those encountered in the control random sample compared to those common among travel survey individuals. For example, the DTW similarity of common land use types of Twitter users and the control sample was found significantly higher (shorter DTW separation) than the average similarity of common land use types of Twitter users and travel survey individuals for all ranks (Welch two sample t-test, p-value < 2.2e-16). These results indicate that there are different individual preferences of where do users engage the most. The results also contradict the implicit semantic coherence assumption, which is used to assign land use types to individual users key locations based on the density of activity (tweeting).

### Temporal coherence of Twitter users

**Fig 4:**
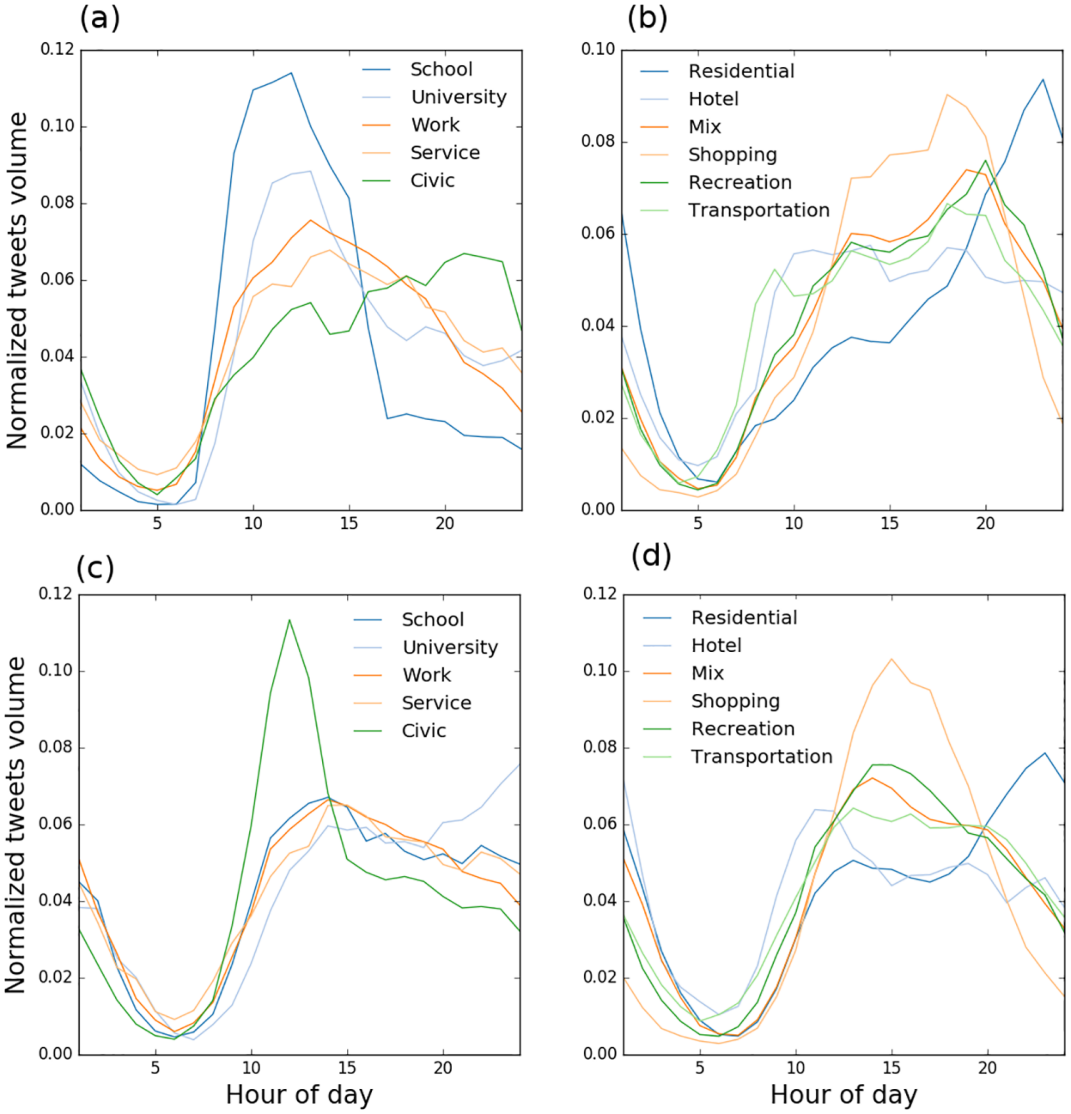
Twitter temporal signatures. A-D: Twitter users’ temporal signatures aggregated by land use type for all users during weekdays (A-B) and weekends (C-D). Weekdays were defined as Mondays to Fridays while Weekends include Saturdays and Sundays. Signatures were normalized by the total number of tweets counts in a land use class to allow comparisons.

Changes in the volume of social media data over the course of a day are sensitive indicators of urban land use [20, 31, 32]. We examined the hourly changes in the number of tweets for all users grouped by land use to identify distinct temporal signatures (Fig 4). We identified four critical times around 7 am, 12 pm, 3 pm and 8 pm based on the abrupt change of volume of tweets. These times were used to distinguish the following categories of land use: 1) Schools with a number of messages that peaks in the morning and drops significantly after 3 pm, 2) Workplaces, where activity peaks in the early morning and remains until 6 pm, 3) Shopping-Recreation, with activity peaks between 6 and 8 pm, and 4) Residential which has a distinct activity peak in the evening (refer to the Methods section). The convolution of these temporal signatures results in a curve with two peaks around lunch and late evening hours, which has been observed in previous studies [33, 34]. However, distinct signatures were found in this study thanks to the high resolution of Chicago land use map and implementing a scalable point in polygon algorithm, which is capable of handling a large volume of tweets [35].

**Fig 5:**
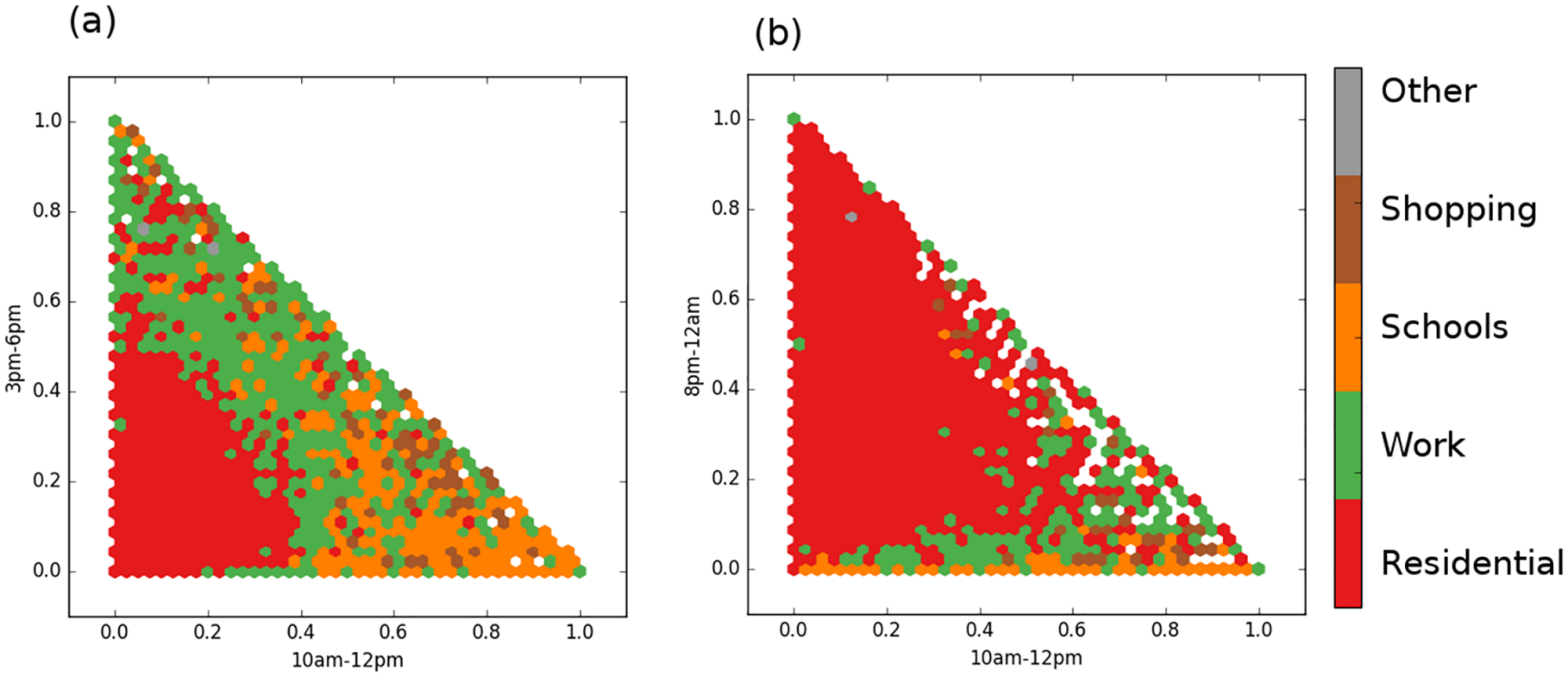
Scatter plots of temporal signatures of individual key locations. A-B: Distribution of individual clusters in a 2D space defined by the temporal activity (percentage of tweets relative to the total number of tweets in the cluster) during different hours of the day. A: morning vs. evening. B: morning vs. afternoon. Clusters with similar land use attributes have a similar distribution of tweets within the twenty-four cycle. Hexagonal binning was used to display the common (mode) land use attribute in each bin.

While previously discussed experiment demonstrated the potential for collecting temporal signatures based on tweets from all users, we wanted to evaluate extracting temporal signatures for individual Twitter users’ key locations. The advantage of characterizing land use at the individual user level is inferring urban land use types at a high spatial resolution, which is comparable to parcel-level maps. We examined the similarity of temporal signatures of individual users’ key locations (temporal coherence scenario) by plotting key locations that have more than twenty tweets in scatter plots, which are defined in terms of the relative number of tweets sent during different periods of the day. Temporal scatter plots (Fig 5) show coherent patterns, where key locations with similar land use types are located next to each other in the feature space. This temporal coherence supports our hypothesis that the timing of tweeting at key locations is indicative of their land use types and consistent among a large number of users.

### Inferring land use from temporal activities of Twitter users

The main objective of this study is to identify reliable clues from Twitter users mobility patterns that help in mapping urban land use. Results in the previous three sections indicate that Twitter users exhibit a preferential return to a few key geographic locations for each user. Furthermore, the temporal patterns of tweeting at these locations are similar for the majority of users and correlated with the locations land use types. In order to achieve our main objective, we evaluated three classification algorithms (a) Random Forest, (b) Decision Tree Classifier and (c) Linear Discriminant Analysis to predict land use types of users’ key locations based on temporal signatures observed at these locations. Classification algorithms were evaluated using a 10 fold cross-validation scheme.

The overall accuracy of the classifications ranged between 0.76-0.77 using three algorithms: Random Forest(0.774), Decision Tree Classifier (0.765) and Linear Discriminant Analysis (0.761). The confusion matrices (Table 1) support the promise of classifying individual users key-locations despite the over-representation of residential land use in the training sample and the variable number of tweets between clusters. The false negative and positive rates indicate that classification algorithms are capable of identifying clusters with distinct temporal changes, such as residential and schools, with a higher accuracy than clusters that are labeled recreation/shopping or work. However, enhancing the quality of classification extends beyond the scope of the current study.

**Table 1:** Confusion matrix of Twitter land use classification.

**Random Forest**			
	Precision	Recall	F-score
*Residential*	0.814	0.979	0.893
*Shopping*	0.422	0.129	0.233
*Schools*	0.614	0.662	0.638
*Work*	0.402	0.17	0.261
**Decision Tree**			
	Precision	Recall	F-score
*Residential*	0.807	0.978	0.888
*Shopping*	0.412	0.075	0.176
*Schools*	0.535	0.613	0.573
*Work*	0.361	0.173	0.25
**Linear Discriminant Analysis**			
	Precision	Recall	F-score
*Residential*	0.804	0.977	0.886
*Shopping*	0.385	0.021	0.09
*Schools*	0.482	0.703	0.582
*Work*	0.369	0.163	0.245

## Discussion

We extracted 508,062 key locations (clusters with four or more unique tweets within a 250 m radius) from the movements of 401,244 unique Twitter users to detect fine-scale spatial heterogeneity of urban land use in the city of Chicago. Unlike previous research, which extracted spatial and temporal Twitter activity collectively for all users, we focused on analyzing individual users behavior at their top tweeted-from locations to extract land use information at a high spatial resolution. We found that the majority of users’ key locations were overlapping with land use parcels indicating the presence of a preferential return among Twitter users. Although density-based clustering algorithms are capable of identifying key locations, their performance could be affected by the compactness of clusters. For example, we found that a number of users were found to be associated with multiple schools or universities. This situation is likely to be the result of splitting irregular and large clusters located in a single parcel and less likely to be related to users relocation given that the average users’ engagement is around a month (Figure 3 in S1 File).

This study also demonstrated the absence of semantic coherence among social media users. We found no generalizable relation between the ranks of a user’s locations, based on the density of tweets, and their land use types. This finding contradicts the heuristics used by the research community to assign land use labels to a user’s key locations assuming that the two most tweeted-from clusters are necessary home and work locations for the majority of users. We found that semantic coherence is present in the travel survey results because of the systematic recording of people’s locations independent of their biases. Our study suggests re-examining algorithms which utilize generic assumptions about the nature of popular locations for users (e.g. top tweeted-from locations) as they vary from one user to another. For example, the most popular location for a Twitter user might be a preferred coffee shop and not necessarily the home or the work locals of this user.

On the contrary, We provided evidence supporting the association of users’ temporal activity and land use type. More importantly, our study provides evidence in favor of the similarity of temporal activities patterns among the majority of Twitter users (temporal coherence). Although, the potential of using Twitter users temporal signatures in urban studies was demonstrated in previous research, extracting signatures was done for a large ensemble of users, which resulted in coarse resolution land use maps [20, 21]. Our investigations demonstrate that temporal information contained in individual clusters are sufficient to train classification algorithms, which is advantageous because fine scale movements of users reveal micro-variability of land use at a scale comparable to parcel-level maps or high-resolution satellite images.

A fundamental assumption underlying the analysis of big data is the consistency of data generation processes. Without big data consistency, it is difficult to train machine learning algorithms and derive accurate predictions. Our study demonstrates that not all aspects of geo-located social media data are consistent. Further research is needed to evaluate the regional consistency of social media signatures across different metropolitan areas [36]. Nevertheless, applying our method allows monitoring of urban land use change at unprecedented resolutions. Our study also contributes to understanding users’ biases in relation to the analysis of human mobility patterns as depicted through the lens of social media.

## Materials and methods

### Datasets

Geo-located tweets were collected over North America using public Twitter streaming API [37] from January 1st, 2013 through February 29th, 2016. We used a bounding box with lower left and upper right corners’ coordinates 41.201577N, -88.707599W, 42.495775N, -87.524535W respectively to filter out the tweets that were posted from outside the city of Chicago. In addition, we also filtered out redundant tweets and tweets without true geographic coordinates. Each tweet in the final dataset contained a geotag and a timestamp.

The land use inventory for Northeastern Illinois is one of the most detailed and updated land use maps of Chicago [26]. The map contains sixty different land use classes and was created using color orthorectified aerial photography captured in April 2010. We re-projected the land use map from the original local projection NAD 1983 State Plane Illinois East FIPS 1201 Feet to WGS84.

We used the Chicago Travel Tracker Household Travel Inventory of 2008 [27], which is a survey conducted over eight counties of the Northeastern Illinois Region. The survey was administered between January 2007 and February 2008, and during this period a total of 32,366 participants were surveyed.

### Twitter data preparation

The number of tweets per unique user was found to follow a heavy tail distribution with a mean of four tweets per user. We restricted our analysis to users with a minimum of four tweets and a maximum speed between any two consecutive tweets of 241 m/s (aircraft speed). Users were further filtered by selecting only those who have at least one cluster with a minimum of four tweets within a 250m radius [25].

The trajectory of each Twitter user is formed from a chronologically ordered list of their geo-located tweets during the study period. For user *i*, the trajectory *T*_*i*_ is defined as [38]:

$$T_{i}=\left\{\left(location_{1},time_{1}\right),\ldots ,\left(location_{n},time_{n}\right)\right\}$$ (1)

Where:

$$\begin{array}{c} \forall 1\leq j\leq k\leq n:{time}_{j}\leq {time}_{k} \end{array}$$

We constructed the semantic trajectories by associating each geo-located tweet with one of the land use types extracted from the land use inventory for Northeastern Illinois [26]. By integrating the land use type to each tweet location, the semantic trajectory of each user is defined as:

$$T_{i}=\left\{\left(location_{1},time_{1},landuse_{l_{1}}\right),\ldots ,\left(location_{n},time_{n},landuse_{l_{k}}\right)\right\}$$ (2)

Where:

$$\begin{array}{c} \forall j\in \left[1,k\right]:l_{j}\in \left[1,60\right] \end{array}$$

After an initial run, we found that a considerable number of geo-located tweets fall on the road network polygons (Table 1 in S1 File) because this class forms a background for other classes (residential, commercial, etc.). Therefore, we decided to reassign geo-located tweets that fall on the road networks to the nearest land use parcel since it is unlikely that streets could count as top-visited locations. Rather, users are expected to be tweeting on the streets in the vicinity of their significant geographic locations (e.g. home) and also to account for inaccurate GPS coordinates.

### Twitter users clusters: Identifying top-visited locations

We applied the DBSCAN clustering algorithm [39] to each unique user trajectory *T*_*i*_ to identify spatial clusters of tweets which are associated with top-visited locations of each user. All the unclustered tweets are labeled as unclassified.

Our analysis yielded 508,062 Twitter user locations in the city of Chicago. The DBSCAN algorithm was selected because it does not require a prior knowledge of the number of key locations (i.e. clusters). We defined a search window of approximately 250 meters (0.0025 degrees) [25] to account for the variability of GPS accuracy between devices, the influence of buildings and walls on GPS accuracy and the fact that top-visited locations are not infinitesimal points on a map. The minimum number of points to form a cluster was selected to be four to ensure that it is a true location and not merely a coincidence.

The clusters were ordered in a descending fashion based on the number of associated tweets. Therefore for each users the frequently visited locations are defined as:

$$\begin{array}{c} FLV_{i}=\left\{Cluster_{1},\ldots ,Cluster_{i_{n}}\right\} \end{array}$$ (3)

Where:

- ∀1 ≤ *j* ≤ *k* ≤ *i*_*n*_ : |*Cluster*_*j*_| ≤ |*Cluster*_*k*_|
- *i*_*n*_ is the number of frequently visited locations of user *i*.

To extract the clusters of each users, we developed a Hadoop code [35] that groups the tweets by their Twitter user id and then calculates the clusters for each user. All unclassified tweets were discarded at this step.

### Measuring cluster spatial uncertainty

We developed a spatial uncertainty index to test the hypothesis that clusters of tweets found in unique Twitter users trajectories overlap spatially with points of interest (land use parcels in our case). The degree of overlap (named here spatial uncertainty) of any identified top tweeted-from cluster, C, was assessed using the ratio of tweets that belong to the dominant parcel with a particular land use type to the total number of tweets.

$$Spatial\;uncertainty\;of\;C=\frac{n}{N},\;\forall C\;\in T_{c}$$ (4)

Where n is the number of geo-located tweets that belong to the dominant parcel in a tweet cluster, C, in a user’s semantic trajectory Tc, and where N is the total number of geo-located tweets in the cluster, C.

### Semantic coherence of Twitter users

The land use inventory for North-eastern Illinois provides an overview of land use types in the City of Chicago at the parcel level. The map has sixty land use classes, which were reclassified to twelve based on a popular activity scheme [40](Table 1 in S1 File). The distinction of urban from rural areas was done using the geographic boundaries of the 2010 Census Urbanized Area. Parcels that fall outside the urban area polygons were excluded from this study.

Tweets clusters of each unique users were sorted in a descending order where rank one is the cluster with the largest number of tweets. The land use type of each cluster of tweets was determined using the most frequent land use label among its tweets and the land use label of each tweet was determined using the land use of the nearest parcel. Clusters were pooled from all users for each rank independently and grouped by their land use types. The number of users in each land use type was normalized by the total number of users in that rank.

A similar analysis was conducted using the Travel Tracker survey data to study the semantic coherence of Chicago residents. The original twenty-five classes of land use/activities (trip purpose field) were reclassified to twelve classes following the same land use scheme, which was used to group the land use map of Chicago (Table 2 in S1 File). The reported activities were sorted for each surveyed individual independently in a descending order based on the duration of each activity, where rank one is the activity with the longest duration. Once sorted, the activities were pooled from all surveyed individuals for each rank independently. The weight of each land use type (number of individuals reported this land use type) was normalized by the total number of individuals in this rank.

Common land use types among Twitter users and travel surveyed individuals were compared by pulling a hundred samples from each dataset. Each sample contained 10,000 random individuals and was used to estimate the weight (percentage) of different land use types in each rank following the same steps described earlier. Missing land use classes from the Travel Tracker survey (i.e. hotels) were omitted from both datasets before normalizing the count to provide a fair comparison. A control group was introduced by taking a hundred samples from the land use map. Each control sample included 10,000 random land use parcels taken from the map without replacement. The control group provided a baseline of expected land use weights in the absence of any individual preference and where it is only controlled by the abundance of land use types in the urban area.

The similarity of common land use weights among Twitter users was compared to those found among the travel survey individuals or in the control group for each rank independently. In this regard, each sample is a vector (1d array) of relative land use weights. The similarity between common land use weights was estimated by calculating the distance matrix between samples from the two data sets (100 samples from each dataset and 10,000 comparisons in total). The similarity distance between any two samples was calculated using the dynamic time warping algorithm implemented in R package dtw [41]. The significance of the difference between land use similarity of Twitter users and the control as well as the land use similarity of Twitter users and travel survey individuals were tested using one-sided Welch’s test under the assumption of unequal variances. All statistical analysis was conducted using the statistical package R [42].

### Characterizing land use based on Twitter temporal signatures

Twitter temporal signatures were estimated by counting the number of tweets in twenty-four-hour bins for each of the twelve land use types separately and then normalize them by the total number of tweets associated with a land use type for all users. Temporal signatures were also estimated for each of the identified users’ key locations with a minimum of twenty tweets. However, the twelve land use types were further aggregated to five types as follows: 1) Residential, 2) Shopping and recreation: shopping, recreation, residential-commercial mix, hotels, and transportation; 3) Schools (k-12 schools); 4) work-civic: offices, service, civic and campuses and 5) other: agriculture, rural, etc. The aggregation was done because of the limited number of tweets usually found in a single key-location.

We compiled a dataset of land use labels and the corresponding hourly temporal signatures for all individual key locations with twenty or more tweets. A 10-fold cross-validation strategy was adopted to evaluate the performance of classifying these key locations. The dataset was split 90% training and 10% for evaluation and we iterated through the evaluation sets for 10 times. Three classifiers (Random forests, Classification trees, and Linear Discriminant Analysis) were trained on the temporal signatures of the tweets key-locations. We evaluated the performance of each classification algorithm using the overall accuracy. In addition, we calculated the confusion matrix for each algorithm by pooling the false positives and false negatives from the 10 folds evaluation. Classification and evaluation of classes were conducted using Python package Scikit-learn [43].

## Supporting information

S1 File Supplementary materials. (PDF)
