# Supplementary material for: Social sensing of urban land use based on analysis of Twitter users’ mobility patterns
Source: PLoS One. 2017 Jul 19;12(7):e0181657. doi: 10.1371/journal.pone.0181657 (PMC5517059; doi:10.1371/journal.pone.0181657)
Supplement: S1 File — (PDF) [file pone.0181657.s001.pdf]

# Social Sensing of Urban Land Use based on Analysis of Twitter Users' Mobility Patterns

Aiman Soliman [1,2,5], Kiumars Soltani [1,4,5], Junjun Yin [1,3,5],
Anand Padmanabhan [1,3,5], Shaowen Wang [1,2,3,4,5]

1 CyberGIS Center for Advanced Digital and Spatial Studies
2 National Center for Supercomputing Applications (NCSA)
3 Department of Geography and Geographic Information Science
4 Illinois Informatics Institute
5 University of Illinois at Urbana-Champaign, Champaign,IL, USA

## Supplementary Materials

### 1. Twitter Data Characteristics

The distribution of the total number of tweets per unique user was found to follow a power-law. The power law distribution suggests that most of the tweets were sent by a limited number of users, while the majority have contributed a fewer messages (Figure 1). The heavy tail distribution of number of tweets per users has implications on the quality of the collected data. First, Twitter users with the largest number of tweets are not necessarily the most mobile or active. Second, if data is extracted over a narrow period (e.g. an hour) it will contain a fewer number of tweets because it is unlikely that all users will contribute at the same time. The distribution of time intervals between tweets in Figure 2 suggests that tweets come in bursts. It is very likely that Twitter users remain active once they already tweeted, but as time passes, it is unlikely that they will post tweets.

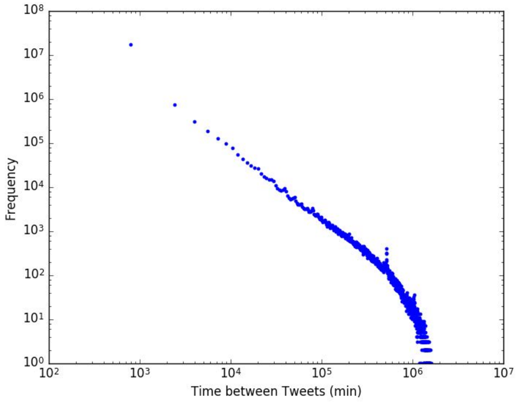


**Figure 1. Distribution of time intervals between consecutive tweets.**

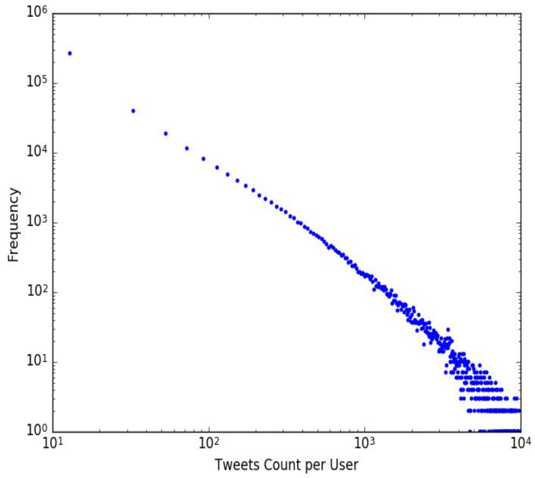


**Figure 2. Distribution of the number of tweets per unique user.**

## 2. Twitter Users Characteristics

We evaluated the engagement of Twitter users during the study period (2013-2016) to measure the representativeness of Twitter data. We calculated the Shannon entropy over the 36 calendar months to estimate the engagement variability. The Shannon entropy of the monthly tweeting behaviour for a particular user is defined as

$$S^k = -\sum_{j=1}^{36} p_k(j) \, log_2 \, p_k(j)$$

where $p_k(j)$ is the probability that the user $k$ has tweeted in the calendar month $j$ .

The maximum expected entropy is 5.170 in the case that a user engages equally during all thirty-six months. While the minimum entropy is 0, which is the case when a user tweets during a single month only. We tested the hypothesis that Twitter users residing in Chicago are likely to tweet during the entire period of three years, which will result in their monthly Shannon entropy distribution concentrated at the maximum entropy value. The results (Figure 3 and 4) indicate that counter to our intuition, the engagement of Twitter users is not homogeneous across the user community. The majority of users engage over a single month as indicated by the large peak at zero entropy. However, the remaining users show a wide range of engagement spreading across the complete entropy range, which indicates a multitude of engagement behavior.

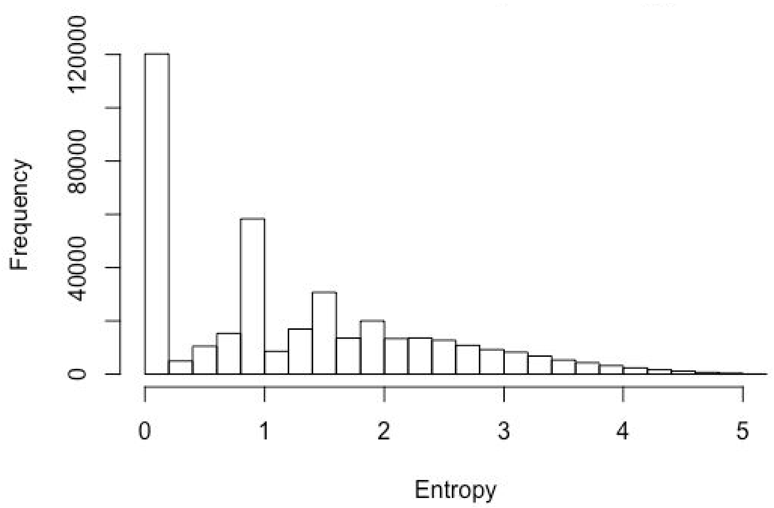


**Figure 3. Monthly temporal entropy; Assuming an equal engagement probabilities across the thirty six calendar months, the minimum entropy is 0, the maximum is 5.170. The large percentage of the user engaged during a single month, the remaining users showed a large variability as indicated by the spread of their temporal entropy across the entire range.**

This heterogeneous engagement would impact the stability of the number of unique Twitter users taken from a random sample especially if collected over a short period (e.g. a single month). The results of the temporal entropy analysis suggest that the number of unique users in a metropolitan area like Chicago varies considerably over a short period because new users are added and old users are dropping on a monthly basis. Therefore, it is likely that a sample obtained over a shorter time period to be biased towards users who are active during this month.

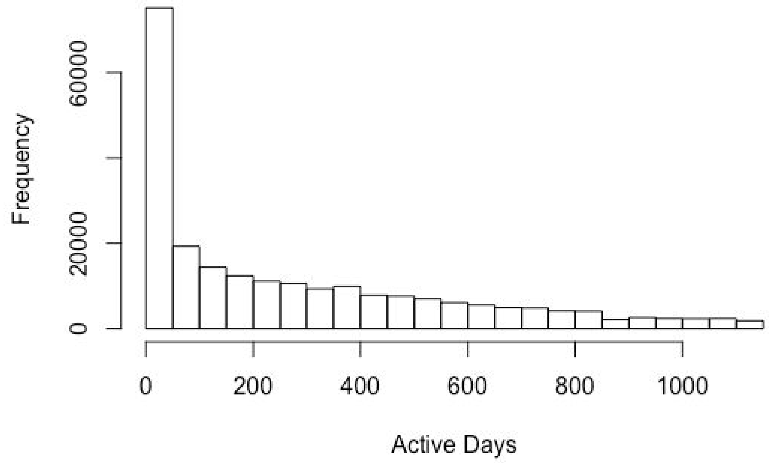


**Figure 4. Distribution of active days for all users during the study period.**

The stratification of Twitter users based on their tweeting behavior would have a strong impact on our study. A previous study of the predictability of Twitter users movements (Jurdak et al., 2015) , conducted in Australia, found that Twitter users have two distinctive subpopulations, a group that maintained low randomness and high regularity, while the other group have a higher randomness and low regularity given the same number of key locations (spatial clusters) and the sequence of the previous visitation. The differences between users could be attributed to their spatial mobility patterns or to the correlation between their tweeting preferences and mobility patterns. If it is the latter case, our findings might be subjected to a population stratification.

We tested the Chicago Twitter populations for the existence of subpopulations by calculating the correlated entropy ($S_{cor}$) that a user visited a frequently visited location given the history of the previous visitations using

$$S^k_{cor} = \sum_{T^*_k \subset T_k} p_k(T^*_k)\, log_2 p_k(T^*_k)$$

where p(T*) is the probability of finding a visitation sequence T* in the trajectory T of the Kth user. The occurrence of repeated visitation sequence was estimated using a Lempel-Ziv algorithm following (Jurdak et al., 2015).

The distribution of the correlated entropy showed a decrease of predictability as the total number of key locations (N) increase (Figure 5).

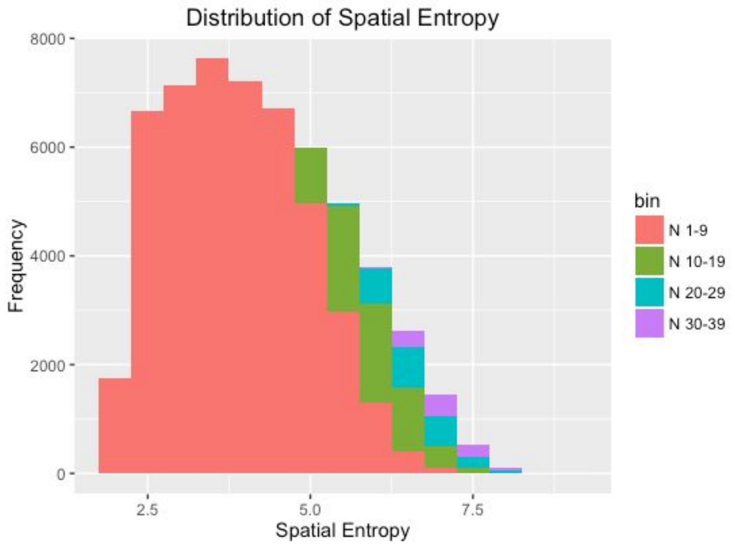


**Figure 5. Correlated entropy for all users with more than 100 tweets per year. The entropy is grouped in four categories with a minimum number of clusters of 1, 10, 20 and 30 respectively. Although the entropy distribution is skewed towards lower value, however, there is no evidence of multimodality.**

Remarkably, there are no clear subpopulations identified based on the entropy distribution compared to the previous study conducted on Twitter users in Australia.  This finding is justified given that it is more likely to find evidence of Twitter users' stratification in a large continent and a large user community than in a metropolitan area like Chicago.

## 3. Twitter Users' Key-Locations/Clusters Characteristics

We conducted analysis on the properties of users clusters (key-locations). Our results show that the number of key locations (clusters) per user follows a heavy tail distribution where the majority of users have limited number of key locations (Figure 6). Similarly, the distribution of active days for each cluster also followed a heavy tail distribution indicating that the majority of clusters remained active for less than fifty days (Figure 7).

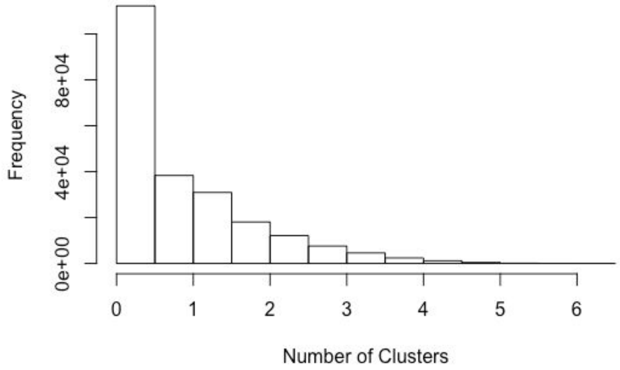


**Figure 6. Log distribution of number of clusters per user**

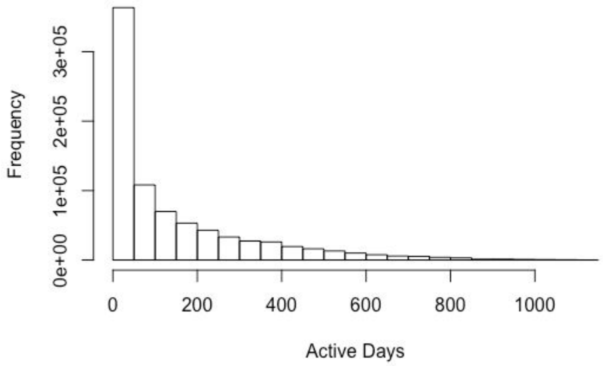


**Figure 7. Distribution of clusters active time**

## 4. Grouping Land Use Types

The original land use types in the parcel-level maps were grouped into twelve major types based on a classification scheme adopted by (Jiang et al, 2012). We added three new categories to the original 9 types, which are hotel, universities, and land use mix. Road network polygons (class 6300) were removed from the analysis.

**Table 1. Grouping of utilization types of the Chicago land use map (CMAP, 2014)**

| Corresponding Land Use | Original Land Use Types of CMAP |
|---|---|
| **Residential** | 1111 Single-Family Detached [RES_SF_DETACHED]<br>1112 Single-Family Attached [RES_SF_ATTACHED]<br>1130 Multi-family [RES_MF]<br>1140 Mobile Home Parks and Trailer Courts [RES_MOBILE]<br>1151 Common Open Space in a Residential Development [RES_COMMON] |
| **Hotel** | 1250 Hotel/Motel |
| **Land Use Mix** | 1215 Urban Mix [COM_URBMIX]<br>1216 Urban Mix w/Residential Component [COM_URBMIXwRES] |
| **Schools** | 1321 K-12 Educational Facilities [INST_EDU_K12] |
| **Universities** | 1322 Post-Secondary Educational Facilities [INST_EDU_HIGHER] |
| **Work** | 1220 Office [COM_OFFICE]<br>1410 Mineral Extraction [IND_MINERAL]<br>1420 General Industrial < 100 000 sq. ft. [IND_GENERAL]<br>1431 Manufacturing/Processing >= 100 000 sq. ft. [IND_MANUF_100K]<br>1432 Warehousing/Distribution >= 100 000 sq. ft. [IND_WAREH_100K]<br>1433 Flex or Indeterminate >= 100000 sq. ft. [IND_FLEX_100K]<br>1450 Storage [IND_STORAGE] |
| **Service** | 1310 Medical Facilities [INST_MEDICAL]<br>1330 Government Administration and Services [INST_GOVT] |
| **Civic Religious** | 1350 Religious Facilities [INST_RELIGIOUS]<br>1360 Cemeteries [INST_CEMETERY]<br>1370 Other Institutional [INST_OTHER]<br>1380 National Laboratory [INST_NATLAB] |
| **Shopping** | 1211 Shopping Malls [COM_MALL]<br>1212 Regional & Community Retail Centers [COM_REGIONAL]<br>1214 Single Large-Site Retail [COM_BIGBOX] |

**Table 1. Continued**

| Corresponding Land Use | Original Land Use Types of CMAP |
|---|---|
| **Recreation** | 1240 Cultural/Entertainment [COM_CULT_ENT]<br>3100 Open Space Primarily Recreation [OS_REC]<br>3200 Golf Course [OS_GOLF]<br>3300 Open Space Primarily Conservation [OS_CONS]<br>3400 Non-Public Open Space [OS_PRIVATE]<br>3500 Trail or Greenway [OS_TRAIL] |
| **Transportation** | 1511 Rail ROW [TCU_ROW_RAIL]<br>1512 Roadway [TCU_ROW_ROAD]<br>1520 Other Linear Transportation with Associated Facilities [TCU_OTH_LINEAR]<br>1530 Aircraft Transportation [TCU_AIR]<br>1540 Independent Automobile Parking [TCU_PARKING] |
| **Other** | 1340 Prison and Correctional Facilities [INST_PRISON]; 1550 Communication [TCU_COMM]<br>1561 Utility Right-of-Way [TCU_ROW_UTIL]<br>1562 Wastewater Treatment Facility [TCU_WWTP]<br>1563 Landfill [TCU_LANDFILL]<br>1564 Other Utility/Waste [TCU_OTH_UTIL]<br>1565 Stormwater Management [TCU_STORMWATER]<br>1570 Intermodal Facility [TCU_INTERMODAL]<br>2000 Agriculture [AG] |

**Table 2. Translating travel survey activities (CMAP, 2008) to equivalent land use type.**

| Corresponding Land Use | Original Travel Survey Activity Types |
|---|---|
| **Residential** | 1 Working at home for pay<br>2 All other home activities<br>21 Visit Friends & Relatives |
| **Hotel** | Not identifiable in survey data |
| **Land Use Mix** | Not identifiable in survey data |
| **Schools** | 5 Attending class<br>6 All other activities at school |
| **Universities** | Not identifiable in survey data |
| **Work** | 3 Work/Job<br>4 All other activities at work<br>11 Work/Business related<br>12 Service Private Vehicle<br>16 Personal Business |
| **Service** | 18 Health Care |
| **Civic Religious** | 19 Civic/Religious Activities |
| **Shopping** | 14 Shopping for major purchases<br>13 Routine Shopping<br>17 Eat meal outside of home |
| **Recreation** | 20 Recreation/Entertainment<br>24 Loop trip |
| **Transportation** | 7 Change type of transportation/transfer<br>8 Dropped off passenger from car<br>9 Picked up passenger<br>10 Other transportation<br>15 Household errands |
| **Other** | 97 Other, specify |

# References

CMAP (2008). Chicago Travel Tracker Household Travel Inventory.

CMAP (2014). Chicago Metropolitan Agency for Planning's 2010 Land Use Inventory for Northeastern Illinois (Chicago, IL).

Jiang, S., Ferreira, J., and González, M.C. (2012). Clustering daily patterns of human activities in the city. Data Min. Knowl. Discov. *25*, 478–510.

Jurdak, R., Zhao, K., Liu, J., AbouJaoude, M., Cameron, M., and Newth, D. (2015). Understanding Human Mobility from Twitter. PLoS ONE *10*, e0131469.
